# 

**Published:** 1893-01

**Authors:** 


### LIST OF CONTRIBUTORS.

---

Anderson, John R.
Archibald, R. A.
Bowker, A. G.
Bowman, E.
Bryden, Williamson.
Butterfield, J. F.
Clement, A. W.
Dinwiddie, R. R.
Dougherty, Wm.
Fisher, J. F.
French, Cecil.
Godfrey, A. H.
Gold, T. S.
Harger, S. J. J.
Harrison, R. H.
Helmer, Jacob.
Hewitt, Sarah Cooper.
Hinebauch, T. D.
Hopkins, Jas. D.
Huidekoper, R. S.
Hunting, W.
Mayo, N. S.
Michener, J. C.
Morris, Claude D.
McCall, Professor.
McFadyean, J.
Parker, John M.
Reynolds, M. H.
Shufeldt, R. W.
Tucker, R. L.
Turner, J. P.
Unvolette, Giuseppe.
Waugh, Jas. A.

147432

### INDEX VOLUME XIV.

### A

PAGE
Anderson, John R. Hydrated Chloral.—Chloral Hydrate. Chloral... ..... 365
Answer to Dr. Shufeldt........ 44
Actinomycosis Bovis or Lump Jaw....... 163
Archibald, R. A., Extirpation of a Tubo-Ovarian Cyst....... 224
Apology (Editorial) ........ 246
Artificial Fecundation ........ 341
Alimentary Canal, malformation of........ 101
Agricultural Shows, Judges and the Veterinarian........ 129
Acute Toxic Anæmia........ 150
A comlpicated case of Parturition........ 79
Army Legislation........ 364

### B

Butterfield, J. F., V. S., Intussusception or Invagination........ 17
Bryden, Williamson, Seedy-toe........ 69
Diseased Cattle........ 105
Bowman, E., a complicated case of Parturition........ 79
Bowker, A. G., malformation of Alimentary Canal ........ 101
Bronchitis in Dogs........ 144
Bovine Tuberculosis........ 235

### C

Comparative Medicine and Veterinary Science, Faculty of ........ 59
Clement, A. W., case of Glanders in Man ........ 65
Report of Tuberculosis Committee ........ 380
Country Roads, some suggestions for better........ 81
Communication—Answer to Dr. Shufeldt........ 44
Ontario Veterinary College........ 46
University Dog Hospital ........ 47
A Rejoinder to Dr. J. P. Turner........ 102
Those Diseased Cattle ........ 105
Corrections........ 181, 322
Columbian Exhibition Horse Show........ 193
California State Veterinary Medical Association........ 276
Cuterebra Emasculator in Dog........ 379
Chicago Veterinary College.—A New Veterinary Degree........ 425
Chloral Hydrate.—Hydrated Chloral. Chloral........ 365

### D

Dinwiddie, R. R., some Texas Fever experiments .......... 1
Actinomycosis Bovis or Lump Jaw .......... 163
Dougherty, Wm., Thermometry .......... 14
Diffuse Melanosis in a Cow .......... 32
Diseased Meats as food .......... 74
Diseased cattle .......... 105
Differential Diagnosis .......... 141
Dogs, Bronchitis in .......... 144

### E

Editorial—Greeting 1893 .......... 41
New York State Veterinary Medical Society .......... 42
Public Highways .......... 107
Pennsylvania State Veterinary Medical Association .......... 182
Apology .......... 246
United States Veterinary Medical Association .......... 247, 342
Veterinary Titles .......... 411
Extirpation of a Tubo-Ovarian Cyst .......... 224

### F

Fastest Time Made by Horses .......... 441
Fisher, J. F., Veterinary Dental Surgery .......... 36
Faculty of Comparative Medicine and Veterinary Science .......... 59
Fungus Hæmatodes in cattle and horses .......... 156
Fistula .......... 323
Fecundation, Artificial .......... 341
French, Cecil, Cuterebra Emasculator in Dog .......... 379

### G

Glanders in man, record of a case of .......... 65
Godfrey, A. H., Horse Show, Columbian Exhibition .......... 193
Gold, T. S., Scouring in Calves .......... 312
Glanders-Farcy and Mallein .......... 370
Graduates 1893:
New York College Veterinary Surgeons .......... 431
Chicago Veterinary College .......... 432
American Veterinary College .......... 434
McGill University, Montreal .......... 435
Iowa Agricultural College .......... 435
Ontario Veterinary College .......... 436
Ohio Veterinary College .......... 439
University of Pennsylvania .......... 440
Harvard University .......... 440

### H

Hewitt, Sarah Cooper, Some Suggestions for Better Country Roads.......... 81
Hunting, W., Mallein as an Aid to the Diagnosis of Glanders............... 87
Huideko[illegible]r, R. S., Agricultural Shows, Judges and the Veterinarian......... 129
The Hackney Horse ........................................ 281
Hopkins, James D., Differential Diagnosis .................................. 141
Helmer, Jacob, Acute Toxic Anæmia.... ................ .......... 150
Horse Show, Columbian Exhibition ........................... .. ..... 193
Hayden, E. D. (Treatment of Wounds by Iatrol)......................... 260
Hydrated Chloral........................ . ...................... 365
Harger, S. J. J., Glanders-Farcy and Mallein................. .......... 370
Hackney Horse (The)........................ ............... 281
Harrison, R. H., New Method of Treating Periodic Ophthalmia............ 306
Hinebauch, T. D., Millet Disease...................... ...........313, 396

### I

Intussusception or Invagination..... ................ .... ........... 17
Illinois State Veterinary Medical Association............. ............56, 278
Indiana Association of Veterinary Graduates....... .... ..............184, 362
Iatrol (Treatment of Wounds by).. .................................. 260

### J

Jennings, Prof. Robert, Obituary...................... .... . .... 280

### K

Keystone Veterinary Medical Association............... ........49, 50, 183
Kansas State Agricultural College............. ........................ 279

### L

Lump Jaw........... ........................................ 163

### M

Montreal Veterinary Medical Association.....................61, 186, 188, 190
Maryland State Veterinary Medical Society...... ........................ 63
Massachusetts Veterinary Association........ ...... ............64, 126, 128
Morris, Claude D., Diseased Meats as Food........... ... ............ 74
Osteo-Porosis................. .................. 397
Mallein as an Aid to the Diagnosis of Glanders....... ............... ... 87
Malformation of Alimentary Canal.............. ......................... 101
Michener, J. C., Open Joints ......................................... 160
Millet Disease.......... ............................ ... ....313, 396

### Mc

McCall, Professor, Is Scarlatina a Bovine Malady......................... 19
McFadyean, J., Mallein as an Aid in the Diagnosis of Glanders ............ 87

### N

New York State Veterinary Medical Society..... .. .......42, 112, 125, 345, 397
New Jersey State Veterinary Society.... ... ........................... 192
New Method of Treating Periodic Ophthalmia........................... 306
Notice to the Veterinarians of the State of New York.................... 413

### O

Ontario Veterinary College............................................ 46
Ontario Veterinary Medical Society.................................... 58
Ontario Veterinary Association........................................ 63
Osteo-Porosis......................................................... 141
Osteo-Porosis......................................................... 397
Open Joints........................................................... 160
Obituary, Prof. Jennings.............................................. 280
Ohio State Veterinary Medical Association............................. 361

### P

Pacing, Time Made by Horses........................................... 441
Parturition, a complicated case of.................................... 79
Public Highways....................................................... 107
Pennsylvania State Veterinary Medical Association..................182, 261
Parker, John M., Scouring in Calves................................... 227
Bovine Tuberculosis................................................... 235
Periodic Ophthalmia, New Method of Treating........................... 306

### R

Records, Fastest Time Made by Horses.................................. 441
Rheumatism in Horses.................................................. 141
Reynolds, M. H., Fistula.............................................. 323
Report of the Tuberculosis Committee.................................. 380
Running, Time Made by Horses.......................................... 441

### S

Scarlatina, a Bovine Malady, is, Professor McCall..................... 19
Shufeldt, Dr., a rejoinder to J. P. Turner............................ 102
Seedy-Toe............................................................. 69
Scouring in Calves.................................................227, 312
Society Proceedings:
Keystone Veterinary Medical Association.........................49, 183, 419
Veterinary Medical Association of New Jersey.......................... 53
Society for the Study of Comparative Psychology in Connection with the Faculty of Comparative Medicine and Veterinary Science of McGill University, Montreal, Canada............ 420
Illinois State Veterinary Medical Association......................56, 278
Ontario Veterinary Medical Society.................................... 58
Faculty of Comparative Medicine and Veterinary Science.............59, 420

Society Proceedings :
Montreal Veterinary Medical Association.......................61, 186
Maryland State Veterinary Medical Society.................... 63
Ontario Veterinary Association ........ ...................... 63
Massachusetts Veterinary Association...............64, 126, 128, 414
United States Veterinary Medical Association................... 109
New York State Veterinary Medical Society.. ..........112, 345, 426
Indiana Association of Veterinary Graduates................184, 362
New Jersey State Veterinary Society........................ ... 192
Pennsylvania State Veterinary Medical Association... ........... 261
California State Veterinary Medical Association................. 276
Kansas State Agricultural College............................. 279
Ohio State Veterinary Medical Association...................... 361

### T

Texas Fever Experiments...................... .................... 1
Thermometry........... .................................. ............ 14
Turner, J. P.......... ....................... .................... 44
Tucker, R. L., Bronchitis in Dogs.. ... ............................ 144
Tubo-Ovarian Cyst, Extirpation of a.... ................ ... ....... 224
Treatment of wounds by Iatrol......... ...... .......... .......... 260
Trotting, Time Made by Horses ........................................ 441
Tuberculosis, Report of Committee............. ....................... 380

### U

Unvolette Giuseppe, Diffuse Melanosis ........... .................... 32
University of Pennsylvania, Veterinary Department........................ 47
United States Veterinary Medical Association. ..................109, 247, 342

### V

Veterinary Dental Surgery............................................ 36
Veterinary Medical Association of New Jersey.......................... 53
Veterinary science and the U. S. Army................................. 102
Veterinarians of the U. S. Army....................................... 364

### W

Waugh, Jas. A., Fungus Hæmatodes in cattle and horses........... ...... 156

### ILLUSTRATIONS, VOL. XIV.

FIG. PAGE.

1. Diffused Melanosis in a Cow.................................... 35
   FIG. I. Melanotic Corpuscles in Cow. 750 diameters.
   FIG. II. Melanotic Corpuscles in Mare. *a.* Fusiform cells invaded with melanosis.
   FIG. III. Section of Melano-Sarcoma in the Cow. 450 diameters. *a.* Binucleated connective tissue cells. *b.* Embryonic cells. *c.* Cells not yet invaded with melanosis.
   FIG. IV. Section of Melano-Sarcoma from the Skin of a Man. *a.* Cells partly invaded with Melanotic Corpuscles. *b.* Embryonic cells. 450 diameters.
2. The University Dog Hospital, University of Pennsylvania, Veterinary Department.................................... 47
3. Actinomycosis Bovis, or Lump Jaw.
   PLATE I. Lower Jaw.................................... 166
4. PLATE II. Bones of the Face.................................... 167
5. PLATE III. Jaw-bone, showing effects of Actinomycosis.......... 169
6. PLATE IV. Actinomycosis Magnified. 500 diameters.......... 170
7. PLATES V and VI. Photo-micrographs.......... 172

Horse Show, Columbian Exhibition.

8. Arab Mare, Aga.................................... 203
9. French Coacher, "Perfection".................................... 206
10. French Coach Colt.................................... 207
11. Cleveland Bay, "Highcliffe".................................... 210
12. "Monte Cristo, Jr.," Kentucky Saddle Horse.................... 220
13. Orloff Trotter, "Ouriadnik".................................... 222

The Hackney Horse.

14. "Fashion".................................... 282
15. "Matchless of Londesboro".................................... 284
16. "Little Wonder".................................... 286
17. "Lady Alice".................................... 288
18. "Belle II".................................... 290

